# Adhesion of the Placenta

**Published:** 1875-10

**Authors:** J. G. Swayne

**Affiliations:** Consulting Physician-Accoucheur to the Bristol General Hospital


### ADHESION OF THE PLACENTA.

BY J. G. SWAYNE, M. D.,

*Consulting Physician-Accoucheur to the Bristol General Hospital.*

Adhesion of the placenta is one of those complications of labor which are especially dangerous in the hands of an unskilful accoucheur. Both the diagnosis and the treatment of it demand from the obstetric practitioner the exercise of the tactus eruditus; and, if this be wanting, the most disastrous consequences may ensue. The patient may die either quickly from hemorrhage, or more slowly but not less surely, from septicæmia. Moreover, adhesion of the placenta is not unfrequently the cause of those frightful cases of malapraxis in midwifery which lead to coroners'

inquests. For instance, an incompetent midwife, either male or female, sets about removing what is supposed to be a retained placenta, and, in the rashness of ignorance, tears away the entire uterus, and perhaps a considerable portion of the intestines. It is most desirable, therefore, that we should ascertain the causes of a complication that may be so hazardous, and use every means in our power to prevent it. But here, it must be confessed, our knowledge is very much at fault; the diseases, both of the fœtus and of the placenta and membranes, are at present veiled in the greatest obscurity. Nor is our knowledge of the pathology of the gravid uterus much more advanced. Most obstetric authorities, who have gone much into the etiology of morbid adhesion of the placenta, have been disposed to attribute it to a subacute inflammatory affection of the lining membrane of the uterus, most probably of a rheumatic character. Others, again, such as the late Sir James Simpson (who has written a very able memoir on "Congestion and Inflammation of the Placenta,") are of opinion that placental adhesion is a result of inflammation of the placenta, placentitis. Sir J. Simpson remarks: "The effusion or secretion of coagulable lymph as a termination of placental inflammation is best known by the effect to which it not unfrequently gives rise, of producing more or less intimate and extensive adhesion between the uterine surface of the placenta and the inner corresponding surface of the uterus, constituting a morbid state of the after-birth that frequently forms a most formidable and dangerous cause of the uterine hemorrhage after the expulsion of the child."

The diagnosis of placental adhesion is a matter of considerable uncertainty, unless the hand be introduced into the uterus, so as actually to ascertain by the touch the nature and extent of the adhesion. Before this is done, we can only form probable conjectures. We may suppose, then, there is morbid adhesion, if, after the child is born, on placing the hand over the fundus uteri, the uterus can be felt contracting repeatedly and even energetically without any apparent result; and this presumption is strengthened if the cord, on gently pulling it and then letting it go, be observed to spring back as if the placenta were tightly held by the uterus, especially if we can feel that there is no constriction of the circular fibres of the uterus to account for this. It should be borne in mind, however, that the presence of irregular contraction of the uterus is no proof of the non-existence of placental adhesion; for in some of the worst cases these conditions cöexist. The introduction of the hand is equally necessary in either case, and soon removes all doubt as to the cause of retention. The existence of hemorrhage proves nothing, as this may accompany retention of the placenta from any cause.

The treatment of adherent placenta should be prompt, because this complication is generally aacompanied with hemorrhage, which may speedily become dangerous. It is never safe to leave such cases long to the unaided powers of nature, or to rely on ergot, styptics, or any kind of drug. The hand of the accoucheur supplies the only safe and effectual remedy. To employ this remedy properly, some coolness and dexterity and a considerable amount of patience are required. The hand has sometimes to be kept in the uterus for half an hour or more before the operation can be completed. In the ordinary obstetric position, the woman lying on her left side, the left hand will be found to be much the most convenient for this purpose; but, to perform the operation properly, both hands should be used. The right hand applied externally over the fundus uteri steadies the uterus, and gives important help to the other hand during these manipulations. If, in addition to placental adhesion, there should be an hour-glass contraction of the uterus, the case is rendered more tedious and difficult; for the circular constriction has first to be overcome before the hand can be passed far enough to separate the adhesions. In such a case, the vagina will probably be found to be loose and capacious, and perhaps filled with clots; whilst the upper portion of it is occupied by a firm fleshy mass, into which the cord is apparently inserted. On making a careful examination, however, by passing up the fingers of the left hand along the cord, which should serve as a guide, this mass is found to be the uterus, tightly contracted around the retained placenta; the circular fibres of the os uteri internum surrounding the cord very closely. This is precisely the condition of things which leads to those horrible blunders which are sometimes committed by ignorant and incompetent practitioners. A practitioner of this sort, for instance, having waited a reasonable time after the birth of child, for the expulsion of the placenta, and finding that this does not take place, and that, moreover, there is some hemorrhage, endeavors to remove the after-birth by tugging at the cord. The only result of this proceeding is, that the cord breaks short off, the hemorrhage is increased. By the breaking of the cord, he loses his guide; and, becoming still more flurried from observing the fresh hemorrhage, he passes up his hand, and feels nothing but the uterus tightly contracted around the placenta. Mistaking this for the latter, he gets his fingers into the hollow of the sacrum behind it, with the object of scooping out the after-birth, which he supposes to be (what the old nurses call) "grown to the side." At last, by means of his nails, he succeeds in separating the posterior wall of the vagina from its attachment to the uterus, and gets his hand into the pouch of Douglas, and, of course, into the peritoneal cavity. Still unaware of his mistake, he grasps the fundus uteri from behind; and, thus obtaining a good purchase, he tears away the entire organ; and then at last, with the appearance of the intestines, the horrible conviction of his blunder dawns upon him.

Such is the manner in which we may reasonably suppose that many of these awful cases of malapraxis take place. I need scarcely remark that such accidents ought to be impossible to any one possessed of the smallest amount of skill and anatomical knowledge.

To pass on, however, to the manipulation necessary in these cases: if the cord be tightly encircled by the os uteri, the constriction should be overcome by insinuating the tips of the fingers into the os in a conical form; whilst the right hand all this time is making counter-pressure upon the fundus uteri, so as to steady that organ. Should these precautions be neglected, the connections between the vagina and the uterus may be put very injuriously on the stretch, especially if the circular fibers of the os oppose much resistance to the introduction of the hand. As the tips of the fingers pass through the os, they should be gradually expanded and separated from one another, until, by sheer fatigue, they overcome the contraction of the uterine fibers, so as to allow the passage of the entire hand into the uterus. If the placenta be attached, as it usually is, to the fundus uteri, or if the uterus be in a flaccid condition, it will be necessary to pass the hand much further than when the placenta is attached lower down, or when the uterus is well contracted. I have sometimes had to pass the hand quite into the epigastric region in search of a retained placenta. As soon as the placenta is arrived at, the fingers should be spread out, taking care not to entangle them in the membranes, until the circumference has already detached; the tips of the fingers should be cautiously inserted between this portion and the inner surface of the uterus, and the placenta gradually peeled off. All this time the right hand, externally applied, steadies the portion of the uterus from which the left hand is detaching the placenta, and enables the accoucheur to estimate the thickness of the uterine walls included between the hands, so that he can avoid digging his nails into the substance of the uterus. There is sometimes considerable danger of such an accident when the adhesions are very firm and close. There is also considerable danger of leaving portions of the placenta behind—a risk that one can readily comprehend in such cases as those described by Dr. Ramsbotham, who states: "I have opened more than one body where a part was left adherent to the uterus, and where, on making a longitudinal section of the organs, and examining the cut edges, I could not determine the boundary line between the uterus and the placenta, so intimate a union had taken place between them." In all such difficult cases, it will be necessary to sever the adhesion by using the finger-nails with a kind of sawing motion from side to side. The tips of the fingers are placed in a line like the edge of a saw, keeping the palm towards the placenta and the knuckles towards the uterus, and the sawing motion is continued very slowly and gradually, until the entire placenta is separated and falls into the hollow of the hand. This proceeding sometimes requires a great deal of patience, and is exceedingly tiring; but the accoucheur should take his time about it, working with both hands, and making his ground sure as he goes on, and not withdrawing his hand with the placenta until he is certain that he has brought away every part of it that can be safely separated. It is very seldom, comparatively, that the adhesions are so firm that this cannot be done. Should this, however, be the case, we have a choice of evils: either to run the risk of causing secondary hemorrhage and septicæmia by leaving portions behind, or of causing metritis from injury to the uterus in bringing them away. For my own part, I think that the last of these two courses is the least dangerous, except in very unusual cases. I have notes of the only two instances in which it was necessary to leave any portion of the consequence behind. Fortunately in both, the pieces were expelled on the third day without having caused any untowaad symptoms, although in one the piece expelled was as large as a hen's egg. Of course, in all such instances, the dangers of septicæmia should be guarded against as much as possible by the frequent use of vaginal injections containing Condy's or other disinfectant fluids.—*British Medical Journal.*

